# State Medical Society of West Virginia

**Published:** 1872-06-15

**Authors:** 


### STATE MEDICAL SOCIETY OF WEST VIRGINIA.

This Society held its annual meeting at Wheeling, June 6th and 7th, 1872. The attendance appears to have been good, the papers and discussions interesting, and the social intercourse pleasant and profitable.

The following are the Officers and Committees for the present year: *President*—Dr. R. H. Cummins. *Vice-Presidents*—Drs. Roemer, Davis, Moore. *Secretary*—Dr. Wm. Dent. *Treasurer*—J. E. Hupp. *Committee on Publication*—Drs. Bates, Hupp, Hildreth and Wm. Dent. *Committee on Necrology*—Drs. Haslett, Berkbile and Roemer. *Committee on New Remedies, Medical Division*—Dr. Jepson; *Surgical Division*—Dr. Pipes. *Essayists*—Drs. Davis and Berry. *Committee on Medical Botany*—First District, Drs. Hildreth and Wiesel; Second, Drs. Bronson and Brownfield; Third, Drs. Roemer and Harris. *Committee of Arrangements*—Dr. Davis and the members of the Society in Parkersburg. *Committee on Climatology and Epidemics*—Drs. Roemer, Harris, Hildreth, Wilson and Ramsey.

The next meeting of the Society is to be held in Parkersburg, the first Wednesday in June, 1873.

DEATH OF A FRENCH ANATOMIST AND PHYSIOLOGIST.—Dr. Liegeois, Vice-Professor at the Paris Faculty, and author of well-known works on anatomy and physiology, recently died, after a brief but painful illness, in the city of Paris.

